# Histoplasmosis as a cause of Addison’s disease and arthritis

**DOI:** 10.1093/rap/rkad057

**Published:** 2023-07-06

**Authors:** Amal Basnet

**Affiliations:** Rheumatology Department, ADK Hospital, Henveiru, Male, Maldives

Key message

- Consideration of infectious mimics of arthritis is crucial in an atypical presentation before commencing immunosuppressant.

Dear Editor, A 21-year-old man presented with complaints of intermittent polyarthritis and recurrent boggy swelling around the wrist and ankle joints for 3 years. He was being treated symptomatically with NSAIDs, with some improvement. He had a history of fatigue, loss of appetite and significant weight loss of 25 kg over 2 years and a history of gradual darkening of the skin. Physical examination of the patient revealed a blood pressure that was marginally low at 90/60 mmHg and BMI of 19.3 kg/m^2^. There was bilateral axillary lymphadenopathy, brownish black pigmentation of the palate (Fig. 1A) and generalized hyperpigmentation of the skin. Abdominal examination revealed an enlarged liver, with liver span of 16 cm, and enlarged spleen, 3 cm below the left costal margin. Musculoskeletal examination revealed multiple tender and swollen joints, with tenosynovitis over the wrist and dorsum of the foot (Fig. 1B–D). Investigations revealed serum an angiotensin-converting enzyme level of 25.8 u/l (reference range 8–65 u/l), serum cortisol (08.00 h) of 17.21 nmol/l (reference range 171–536 nmol/l), serum adrenocorticotrophic hormone of 488 pg/ml (reference range 5–60 pg/ml) and CRP of 10.8 mg/l (reference range 0–10 mg/l). However, RF, anti-CCP antibody, ANA, Mantoux test, anti-HIV antibody, hepatitis B surface antigen and anti-HCV antibody tests were all negative. Peripheral lymph node biopsy was reported as having naked granulomas, consistent with sarcoidosis. Contrast-enhanced CT of the thorax and abdomen showed bilaterally enlarged adrenals and moderate hepatosplenomegaly, with no pulmonary or mediastinal involvement (Fig. 1E). Contrast-enhanced MRI of the ankles showed mild joint effusion, with enhancing synovial thickening and tenosynovitis of the extensor digitorum tendons (Fig. 1F). Synovial biopsy from the ankle showed acute on chronic synovitis. Biopsy of the adrenal demonstrated ill-formed granulomas and yeast forms of *Histoplasma*. The patient was started on cortisone replacement (hydrocortisone tablets at a dose of 10, 5 and 2.5 mg at 08.00, 14.00 and 20.00 h, respectively) and oral itraconazole for 1 year. The tenosynovitis and arthritis improved over a period of 1 month without the need for additional anti-inflammatory drugs. The patient had resolution of fatigue and achieved a weight gain of 15 kg over a period of 1 year. Cortisone treatment was continued for >1 year owing to adrenal insufficiency. There was no further recurrence of joint symptoms after treatment with itraconazole and hydrocortisone.

**Figure 1.**
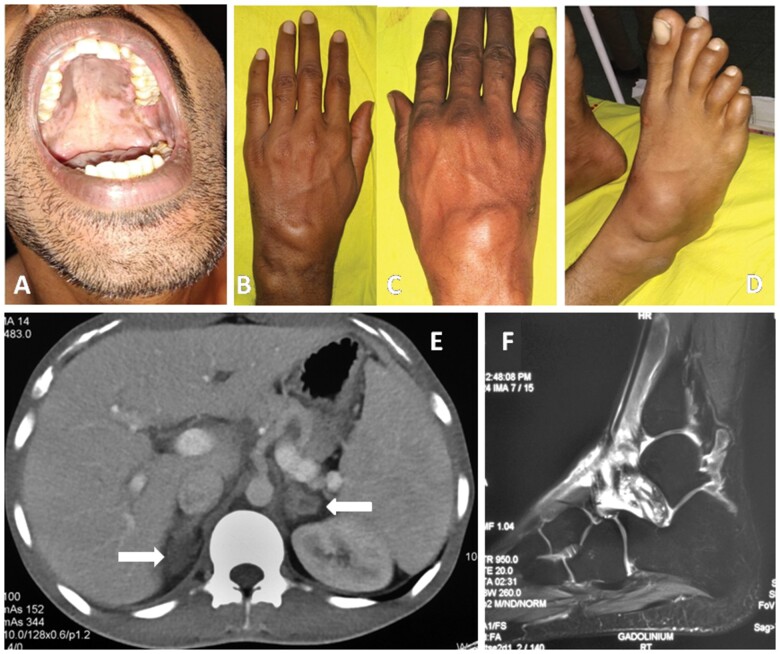
Clinical presentation and diagnostic evaluations. (**A**) Brownish black pigmentation of the palate. (**B**) Tenosynovitis of extensor tendons over left wrist joint. (**C**) Tenosynovitis of extensor tendons over right wrist joint. (**D**) Tenosynovitis of flexor tendons over right ankle joint. (**E**) CT showing bilateral enlarged and hypodense adrenals. (**F**) MRI post contrast images of the ankles showing mild joint effusion with enhancing synovial thickening with tenosynovitis of extensor digitorum tendons

*Histoplasma* is a dimorphic fungus that exists as a mycelium in soil contaminated with droppings from birds and bats. It enters the body by inhalation of spores and can invade the reticuloendothelial system via macrophages. In most cases, it resolves with asymptomatic pulmonary infection, but in some cases it can produce chronic pulmonary disease or disseminated disease. Disseminated disease can involve the liver, spleen, lymph nodes, bone marrow and adrenal glands and has been described in both immunocompetent and immunocompromised hosts [1].

Adrenal histoplasmosis occurs from haematogenous spread of infection. Bilateral extensive destruction of the adrenal glands results in adrenal insufficiency, which occurs in 5–71% of adrenal histoplasmosis. It commonly manifests with fever, fatigue and weight loss, but sometimes with severe adrenal insufficiency, which can be fatal [2]. The mortality rate in untreated disseminated histoplasmosis is as high as 80–100% and decreases to <25% with adequate treatment [3]. In evaluation of bilateral adrenal involvement, the other differentials to be considered besides histoplasmosis include tuberculosis, primary or metastatic malignancy, non-Hodgkin’s lymphoma, sarcoidosis, adrenal haemorrhage and other fungal infections. The disease can be diagnosed with histopathological examination of the involved tissue or by tissue culture, antigen detection, serology and molecular diagnosis by PCR [1]. Adrenal histoplasmosis should be treated like disseminated histoplasmosis, with itraconazole 200 mg twice daily for patients who are not severely ill and with amphotericin B followed by oral itraconazole for severe infection [2].

Rheumatological manifestations of *Histoplasma* in the form of erythema nodosum, arthralgia or arthritis, tenosynovitis, panniculitis and carpal tunnel syndrome have been reported. The articular involvement can be additive symmetrical polyarthritis, oligoarthritis or monoarthritis [4, 5]. True *Histoplasma* arthritis (presence of *Histoplasma* in the joint) is a rare phenomenon, and most of the articular manifestation is secondary to the hypersensitivity reaction to *Histoplasma* antigen present elsewhere in body. The presence of culture-negative joint effusion, the response of the arthritis to anti-inflammatory agents and the coexistence of other features of hypersensitivity in the form of erythema nodosum, pleuritis and pericarditis supports this theory. Histopathological examination of the true *Histoplasma* synovitis has shown necrotizing granulomatous synovitis, with organisms typical of *Histoplasma capsulatum* within the phagocytic cells. Synovial biopsy of the reactive joints has revealed chronic non-specific inflammation, with no evidence of granulomata, which was also true in the present case [4, 6]. Both sarcoidosis and histoplasmosis can present with similar findings of erythema nodosum and splenomegaly, diffuse pulmonary infiltrates, mediastinal lymphadenopathy and elevation of hepatic enzymes, serum angiotensin-converting enzyme and calcium levels. Lymph node biopsy reveals non-caseating granulomas in both [5, 7]. Recommended treatment for rheumatological manifestations in histoplasmosis includes NSAIDs for mild cases and prednisone [0.5–1.0 mg/kg daily (maximum, 80 mg daily) in tapering doses over 1–2 weeks] for severe cases [8].

## Data Availability

The data underlying this article are available in the article.
